# Transcranial Magnetic Resonance-Guided Focused Ultrasound in X-Linked Dystonia-Parkinsonism

**DOI:** 10.3390/life11050392

**Published:** 2021-04-26

**Authors:** Roland Dominic G. Jamora, Wei-Chieh Chang, Takaomi Taira

**Affiliations:** 1 Division of Adult Neurology, Department of Neurosciences, College of Medicine and Philippine General Hospital, University of the Philippines Manila, Manila 1000, Philippines; 2 Section of Neurology and Movement Disorder Service, Institute for Neurosciences, St. Luke’s Medical Center, Global City 1635, Philippines; 3 MR-Guided Focused Ultrasound Center, Chang Bing Show Chwan Memorial Hospital, Changhua County 50529, Taiwan; changweichieh@gmail.com; 4 Department of Neurosurgery, Chang Bing Show Chwan Memorial Hospital, Changhua County 50529, Taiwan; 5 Department of Neurosurgery, Tokyo Women’s Medical University, Tokyo 162-86666, Japan; ttaira@twmu.ac.jp

**Keywords:** X-linked dystonia-parkinsonism, XDP, pallidothalamic tractotomy, transcranial magnetic resonance-guided focused ultrasound, XDP-MDSP scale

## Abstract

X-linked dystonia-parkinsonism (XDP) is a neurodegenerative condition found among males with maternal ancestry from Panay Island, Philippines. The treatment options are limited. We report on our experience of three XDP patients who underwent transcranial magnetic resonance-guided focused ultrasound (tcMRgFUS) pallidothalamic tractotomy. The three patients were all genetically confirmed XDP, with a mean XDP-Movement Disorder Society of the Philippines (MDSP) Scale score of 68.7/200. All patients were on stable doses of their oral medications and their last botulinum toxin injection was 12 months prior to study. Two patients complained of moderate to severe arm pain 2–7 months after the procedure. There was an overall improvement in the XDP-MDSP Scale score of 36.2% (18.7 vs. 15) at 6 months and 30.1% (68.7 vs. 45.5) at 1 year. Notably, there was worsening of the nonmotor subscale (part IIIB, nonbehavioral aspect) by 350% at 1 year. While these numbers are encouraging, there is a need to do a larger study on the safety and efficacy of tcMRgFUS on XDP.

## 1. Introduction

X-linked dystonia-parkinsonism (XDP) is a disabling neurodegenerative condition with motor and nonmotor symptomatology [1,2]. The treatment of XDP has always been unsatisfactory compared to other movement disorders. The use of oral medications has never been absolutely effective [3,4]. Likewise, botulinum toxin is used but is limited by its duration of efficacy and cost [5,6]. The efficacy of pallidal deep brain stimulation (DBS) among XDP patients has been reported [7,8,9]. Recently, the use of magnetic resonance (MR)-guided focused ultrasound for XDP has been reported [10]. Thus, we aimed to report on our experience with transcranial MR-guided focused ultrasound (tcMRgFUS) pallidothalamic tractotomy in three XDP patients.

## 2. Case Reports

*Case 1:* A 31-year-old male with genetically confirmed XDP presented with a 2-year history of increased eyeblinking, followed by smaller handwriting. The following year, he experienced involuntary neck twisting. On examination, there was jaw opening dystonia, right torticollis, truncal hyperextension, flexion of the right toes, and left leg hip flexion when seated, and difficulty ambulating. He has moderate to severe dysarthria, mild masked facies, rigidity (neck and upper extremities, moderate bradykinesia, and gait difficulty). His baseline total XDP-Movement Disorder Society of the Philippines (MDSP) Scale score was 46/200 (I—13/44; II—20/76; IIIA—0/20; IIIB—1/20; IV—12/40) (see Table 1). His cranial MR imaging (MRI) showed symmetric linear signal abnormalities involving both lateral putaminal regions and mild bilateral caudate head atrophy. His medications were clonazepam 5 mg/day, baclofen 30 mg/day, and biperiden 6 mg/day, with a reported relief of about 50%. His last botulinum toxin (BoNT) injection was 12 months prior to the left pallidothalamic tractotomy. 

*Case 2:* A 32-year-old male with genetically confirmed XDP presented with a 4-year history of frequent eye blinking and abdominal contractions, followed by left foot dorsiflexion. A year later, he developed truncal flexion and dysphagia. He eventually had to use a cane for ambulating, progressing to using a wheelchair. On examination, there was jaw opening dystonia, blepharospasm, laryngeal dystonia, left torticollis, flexion of the trunk, and left leg extension associated with difficulty in ambulating. He has mild dysarthria, rigidity (neck and all extremities), moderate to severe bradykinesia, and gait difficulty. His baseline total XDP-MDSP Scale score was 124/200 (I—34/44; II—36/76; IIIA—3/20; IIIB—15/20; IV—36/40) (see Table 1). His cranial MRI showed symmetric linear signal abnormalities involving both lateral putaminal regions and moderate bilateral caudate head atrophy. His medications were clonazepam 4 mg/day and zolpidem 10 mg/day, with a reported relief of about 60%. His last botulinum toxin (BoNT) injection was 12 months prior to the right pallidothalamic tractotomy. 

*Case 3:* A 38-year-old male with genetically confirmed XDP complained initially of eye sensitivity to bright lights for about 2 years. This was followed by involuntary mouth opening and pain on his right hand with stiffness on his left hand. On examination, it was found that he had jaw opening dystonia, left torticollis, and truncal flexion. He has mild masked facies, rigidity (neck and the left extremities), mild bradykinesia in the hands, and a slightly stooped posture. His baseline total XDP-MDSP Scale score was 36/200 (I—9/44; II—13/76; IIIA—0/20; IIIB—3/20; IV—10/40) (see Table 1). His cranial MRI showed bilateral symmetric linear signal abnormalities involving the lateral putaminal regions and mild bilateral caudate head atrophy. His medications were clonazepam, 6–8 mg/day, and biperiden, 8 mg/ day, with a reported relief of about 75%. His last botulinum toxin (BoNT) injection was 12 months prior to the left pallidothalamic tractotomy. 

All patients had neurocognitive and neuropsychiatric clearances prior to the procedure. All patients underwent tcMRgFUS pallidothalamic tractotomy at the Show Chwan Memorial Hospital in Changhuan, Taiwan. This was approved by the Institutional Review Board (SCMH IRB No. 1080201). 

### Procedure

Several papers have been published on the procedure for MRgFUS thermoablation [11,12,13,14,15,16]. The operations were performed in a 1.5T MRI system (GE Optima MR450, USA) using the ExAblate Neuro device (InSightec, Haifa, Israel). Additional care was taken in head immobilization by fixation as our patients have severe cervical dystonia.

The pallidothalamic tract (PTT) was chosen for tractotomy. It was located at the midcommissural line in the anteroposterior direction, 10.0 mm lateral to intercommissural line (ICL) in the mediolateral direction, 7.5 mm to the thalamo-ventricular border, and 1.5 mm below to the intercommissural line in the dorsoventral direction. The center of the PTT target was adjusted by about 3 mm lateral to the mamillo-thalamic tract (MTT) and superior and medial to the medial subthalamic nucleus. The second target was 1 mm posterior, superior, and lateral to the first target.

**Figure 1:**
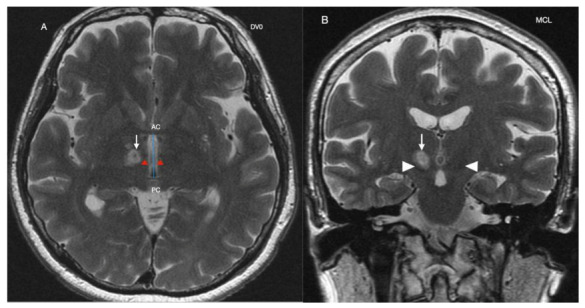
Cranial MRI of Case 2 at day 1 post-pallidothalamic tractotomy. (**A**): Axial cuts at DV0 showing the right pallidothalamic tract target lesion (white arrow) on postoperative day 1. The intercommissural line (blue line) is the distance between the anterior commissure (AC) and the posterior commissure (PC). Red arrows point to the mamillo-thalamic tract. (**B**): Sagittal scan at midcommissural line (MCL). White arrow is target lesion. Arrow heads are the subthalamic nuclei.

The average in-table operation time was 176.8 ± 40.6 min (range, 134–240 min). The mean maximum energy reached 30,750.25 ± 6246.06 J (range, 20,096–35,731 J) with a mean maximum temperature of 58.8 ± 3.1 °C (range, 54–62 °C). The temperature was also monitored in the MTT during sonications. Figure 1 shows the PTT lesion in Case 2, taken one day after the operation.

The patients were awake and responsive during the whole procedures with full physiologic monitoring. They were examined by movement disorder neurologists for treatment effects and adverse events after each sonication. The operations were completed with the improvement of dystonia symptoms, as assessed by the neurologists.

Case 2 experienced claustrophobia and propofol 1%, 10 mg/mL, was intravenously injected at 0.005 mg/kg/min intermittently. The patient was awakened after each sonication. The rest of the patients were not given any sedative agents during the procedures. The patients’ hospitalization was 10 days for preoperative planning and monitoring for complications before they flew back to Manila, Philippines. 

Two months after the procedure, Case 1 started experiencing right arm pain, with a Visual Analogue Scale Score (VAS) of 6–8/10, which was persistent and occasionally relieved with tramadol. For Case 3, he also started to complain of right arm pain, with a VAS 8/10 at 7 months after the procedure, partially relieved by tramadol. 

There was an overall improvement in the XDP-MDSP Scale score of 30.1% (68.7 vs. 48) at 6 months and 33.8% (68.7 vs. 45.5) at 1 year (see Table 1). It should be noted that the 9-month and 12-month follow ups were lacking data from a patient. Looking at the individual subscales, at the 6th month of observation, there was an improvement in part I (dystonia) of 19.7% (18.7 vs. 15), part II (parkinsonism) of 43.5% (23 vs. 13), part IIIA (behavioral nonmotor) of 89% (3 vs. 0.3), and part IV (activities of daily living) of 36.2% (19.3 vs. 12.3). However, there was worsening of the part IIIB (nonbehavioral nonmotor) by 200% (1 vs. 3). The dystonia and activities of daily living scores at 9 months follow up were noted to have worsened.

At 12-month follow up, the improvement was likewise seen in all subscales: 43.8% (18.7 vs. 10.5) for part I, 34.7% (23 vs. 15) for part II, 66.7% (3 vs. 1), and for part IV, 30% (19.3 vs. 13.5), except for part IIIB. At this time, the worsening noted for part IIIB was at −350% (1 vs. 4.5).

## 3. Discussion

The management of XDP is still very limited. There are no effective oral medications available [3,4]. Chemodenervation with botulinum toxin helps but is limited by the cost [5,6]. Pallidal DBS seems to be the best option as of now, with very significant improvement of the dystonia and a variable response of the parkinsonism symptoms [7,8,9]. However, not everyone can undergo DBS due to its cost [6] and possibly due to its invasive nature.

One possible option is the use of tcMRgFUS. This has been approved for other movement disorders such as essential tremors and Parkinson’s disease (PD) [16,17]. It has also been used in a patient with musician’s dystonia [18]. An abstract on the use of tcMRgFUS for XDP is available [10]. Moreover, a clinical trial is underway looking on the efficacy of tcMRgFUS (Vim thalamus or the globus pallidus interna) in patients with treatment-refractory symptoms of movement disorders (essential tremors, Holme’s tremor, PD, dystonia, Wilson’s disease, Huntington’s disease, tardive dyskinesias, and orofacial dyskinesia) (ClinicalTrials.gov Identifier: NCT02252380).

Our patients had tcMRgFUS pallidothalamic tractotomy. The globus pallidus internus is connected to the ventroanterior and ventrolateral parts of the thalamus by the PTT [11]. A lesion in the PTT improves the dystonia by interrupting the cortico-basal ganglia-thalamo-cortical circuit through the modulation of the pallidal efferents to the thalamus [11,19]. 

However, unlike other reports on pain relief, two of our patients experienced severe pain over their right arm months after the procedure. The pain can be described as a central pain syndrome, which usually results from damage to any structure along the spino-thalamo-cortical pathways that convey pain and temperature information and is associated with abnormal inhibitory regulation of the posterior thalamus by the zona incerta [20]. Maldaptive plasticity, brought about by the changes in the connections due to the pallidothalamic tractotomy might cause the pain [20]. 

This pain contributed to the marked worsening (−350% at 12 months) seen in part IIIB of the XDP-MDSP Scale that contributed to the smaller overall improvement seen in our patients. Had the traditional scales (Burke-Fahn-Marsden Dystonia Rating Scale (BFMDRS) and the Unified Parkinson Disease Rating Scale (UPDRS)) been used, we would not have been able to account for these nonmotor symptoms. These nonmotor symptoms are an important determinant of quality of life and disability in movement disorder patients [2]. The XDP-MDSP Scale was developed for XDP patients, using existing scales, including the BFMDRS and UPDRS as the “gold standards” [21]. Moreover, the XDP-MDSP scale has a subscale on nonmotor features (behavioral and nonbehavioral aspects, parts IIIA and IIIB, respectively) and activities of daily living (part IV).

Among patients who had DBS (range of follow up: 6–84 months), the reported improvement in the dystonia (using the BFMDRS) was between 3 and 100%, with worsening of the scores in some patients (7–21%). For the parkinsonian symptoms, using the UPDRS, the improvement was lower at 5–81%, with worsening in some patients of up to 121% [7]. While these DBS results cannot be directly compared with the tcMRgFUS results as a different scale was used, regarding the XDP-MDSP Scale, we can see that in both instances there was an overall improvement in the dystonia and, for a smaller percentage, the parkinsonism. The 12-month data are encouraging, with improvements noted in the different subscales (dystonia, parkinsonism, behavioral nonmotor, and activities of daily living) except for the nonbehavioral nonmotor part (sleep disturbance, pain and other sensations, bladder incontinence, fatigue, saliva and drooling). The biggest improvement was seen in the behavioral nonmotor part (cognition, apathy, anxiety, depression, and irritability and aggression) at 66.7%, followed by dystonia (43.8%), and parkinsonism (34.7%). Indeed, this proved that the nonmotor aspects of the disease are important [2]. 

One advantage of the tcMRgFUS is its noninvasive approach [19]. This also removes the need for repeated patient follow up for DBS programming. We also need to take note of some complications noted with tcMRgFUS thalamotomy, such as sensory and gait disturbance [22]. An inherent limitation of the procedure is its unilaterality. There are no reports on staged campotomy for other types of dystonia, unlike in PD, dystonic camptocormia, and Meige’s syndrome [22,23,24,25]. Another factor to consider is the progression of the disease itself. Unfortunately, we do not have any data on the rate of progression of XDP as well as how it will affect the parkinsonism phase of the disease. Despite these limitations, these cases showed that tcMRgFUS may improve the patient’s XDP-MDSP score, except for the nonbehavioral symptoms at 1-year follow up. There is a need to conduct a controlled study on the long-term safety and efficacy of tcMRgFUS pallidothalamic tractotomy in XDP patients. 

## Figures and Tables

**Table 1:** Summary of XDP-MDSP Scale scores before and after treatment with tcMRgFUS pallidothalamic tractotomy.

XDP-MDSP Scale	Case 1	Case 2	Case 3	Average	% Improvement Compared to Baseline
**Baseline**					
I—Dystonia	13	34	9	18.7	
II—Parkinsonism	20	36	13	23	
IIIA—Behavioral	0	3	0	3	
IIIB—Nonbehavioral	1	15	3	1	
IV—Activities of daily living	12	36	10	19.3	
Total	46	124	36	68.7	
**1 week**					
I—Dystonia	6	30	6	14	25.1
II—Parkinsonism	14	26	13	17.7	23.0
IIIA—Behavioral	0	1	0	0.3	90.0
IIIB—Nonbehavioral	0	6	2	2.7	−170.0
IV—Activities of daily living	6	33	4	14.3	25.9
Total	26	96	26	49.3	28.2
**1 month**					
I—Dystonia	5	27	5	12.3	34.2
II—Parkinsonism	15	29	13	19	17.4
IIIA—Behavioral	0	1	0	0.3	90.0
IIIB—Nonbehavioral	0	6	1	2.3	−130.0
IV—Activities of daily living	6	30	4	13.3	31.0
Total	26	93	24	47.7	30.5
**6 months**					
I—Dystonia	7	28	10	15	19.7
II—Parkinsonism	19	23	7	13	43.5
IIIA—Behavioral	0	1	0	0.3	89.0
IIIB—Nonbehavioral	1	6	2	3	−200.0
IV—Activities of daily living	5	30	2	12.3	36.2
Total	33	88	23	48	30.1
**9 months**					
I—Dystonia	14	25	NA	26.5	−41.7
II—Parkinsonism	20	23	NA	21.5	6.5
IIIA—Behavioral	0	1	NA	0.5	83.3
IIIB—Nonbehavioral	3	6	NA	4.5	−350.0
IV—Activities of daily living	13	30	NA	21.5	−11.4
Total	50	85	NA	67.5	1.7
**12 months**					
I—Dystonia	11	NA	10	10.5	43.8
II—Parkinsonism	23	NA	7	15	34.7
IIIA—Behavioral	1	NA	1	1	66.7
IIIB—Nonbehavioral	4	NA	5	4.5	−350.0
IV—Activities of daily living	17	NA	10	13.5	30.0
Total	56	NA	35	45.5	33.8

NA: not available.

## Data Availability

The data presented in this study are available on request from the corresponding author.
